# Clinical Outcomes and Treatment Characteristics of Seizure Threshold-Titrated Electroconvulsive Therapy in Schizophrenia: A 3.5-Year Retrospective Naturalistic Course-Level Study

**DOI:** 10.3390/jcm15197628

**Published:** 2026-10-01

**Authors:** Claudia Elena Anghel, Ciprian Băcilă, Bogdan Ioan Vintila, Monica Cornea, Andreea Grama, Andrei Lomnasan, Iulian Roman-Filip, Laura Stef, Bălan (Negrău) Cristina Ioana, Corina Roman-Filip

**Affiliations:** 1 Faculty of Medicine, Lucian Blaga University of Sibiu, 550024 Sibiu, Romania; claudia.anghel@ulbsibiu.ro (C.E.A.); bogdan.vintila@ulbsibiu.ro (B.I.V.); corina.roman@ulbsibiu.ro (C.R.-F.); 2 “Dr. Gheorghe Preda” Clinical Psychiatry Hospital of Sibiu, 550082 Sibiu, Romania; moni.sipos@ymail.com (M.C.); andreeamaria.grama@ulbsibiu.ro (A.G.); andreilomnasan96@gmail.com (A.L.); 3 Neuroscience Scientific Research Collective, 550082 Sibiu, Romania; 4 County Clinical Emergency Hospital of Sibiu, 550245 Sibiu, Romania; 5 Doctoral School, “George Emil Palade” University of Medicine, Pharmacy, Science and Technology, 540142 Târgu Mureș, Romania; iulian_roman2009@yahoo.com; 6 Department of Dental Medicine and Nursing, Faculty of Medicine, Lucian Blaga University, 550169 Sibiu, Romania; laura.stef@ulbsibiu.ro; 7 Dental Medicine Research Center, Faculty of Medicine, Lucian Blaga University, 550169 Sibiu, Romania; 8 Bihor County Clinical Emergency Hospital, 410169 Oradea, Romania; cristina.negrau13@gmail.com

**Keywords:** electroconvulsive therapy, treatment-resistant schizophrenia, seizure threshold, positive psychotic symptoms, treatment response, global functioning

## Abstract

**Background**: Electroconvulsive therapy (ECT) is used as an augmentation strategy in treatment-resistant schizophrenia, but real-world data on individualized seizure threshold-guided treatment remain limited. This study evaluated clinical outcomes of seizure threshold-titrated ECT and described seizure expression and technical treatment parameters during acute ECT courses. **Methods**: A retrospective naturalistic study included acute ECT courses performed between March 2022 and December 2025 at the ECT Department of the Dr. Gh. Preda Clinical Psychiatric Hospital, Sibiu, Romania. We assessed clinical outcomes at baseline and post-treatment using the PANSS Positive Subscale (PANSS-P), Global Assessment of Functioning (GAF), and Mini-Mental State Examination (MMSE). ECT was delivered using individualized seizure threshold titration alongside stable psychotropic treatment. Electrode placement, stimulation parameters, and motor and electroencephalographic (EEG) seizure characteristics were recorded throughout the treatment course. **Results**: Seventeen acute ECT courses with paired assessments were analyzed. Median PANSS-P scores decreased from 45 (IQR, 39–48) at baseline to 16 (IQR, 14–20) following ECT (*p* < 0.001), with a Hodges–Lehmann estimated paired reduction of 26 points. Median GAF scores increased from 44 (IQR, 40–45) to 60 (IQR, 60–65), while median MMSE scores changed from 26 (IQR, 26–28) to 29 (IQR, 28–30). Motor and EEG seizure durations varied substantially across courses and sessions. Right unilateral and bilateral electrode placements were used with similar frequency. **Discussion**: Our results align with the existing literature on ECT as an augmentation strategy for persistent positive symptoms in treatment-resistant schizophrenia. Improvement in positive psychotic symptoms was accompanied by better global functioning, while the MMSE detected no deterioration in global cognitive performance. The considerable variability in motor and EEG seizure durations further suggests that seizure duration alone may provide limited information regarding clinical improvement. **Conclusions**: In this retrospective naturalistic cohort, individualized seizure threshold-titrated ECT alongside stable pharmacotherapy was associated with substantial improvement in positive psychotic symptoms and global functioning, without detectable deterioration in global cognitive performance. Given the small sample, the observational design, and absence of a control group, these findings remain preliminary and require confirmation in larger prospective controlled studies.

## 1. Introduction

Schizophrenia is a chronic and severe psychiatric disorder with marked clinical heterogeneity [1]. According to the World Health Organization, it affects approximately 23 million people globally, representing about 0.29% of the world’s population [2]. Despite its relatively low prevalence, schizophrenia is one of the major contributors to years lived with disability because of its substantial impact on cognitive, social, and occupational functioning [3]. Patients with schizophrenia have a life expectancy that is, on average, 15–20 years shorter than that of the general population, mainly due to cardiovascular diseases, metabolic disorders, and suicide [4]. Moreover, the WHO estimates that more than two-thirds of individuals with psychosis worldwide do not have access to specialized mental health services, emphasizing the considerable global treatment gap [2].

Owing to its early onset and long-term functional consequences, schizophrenia is considered one of the leading causes of disability among young adults [5,6]. The disorder has a profound impact not only on mental health but also on social, occupational, and daily functioning, resulting in substantial impairments in patients’ quality of life [7,8,9]. Beyond its psychiatric manifestations, schizophrenia is associated with increased premature mortality [5,10,11], a heightened risk of suicide [5,11,12,13,14], a substantial burden of somatic comorbidities [5,15,16], and considerable socioeconomic costs [17,18]. Therefore, schizophrenia continues to represent a major public health issue. Although important advances in antipsychotic pharmacotherapy have been achieved in recent decades, a significant proportion of patients still experience persistent psychotic symptoms, highlighting the need to identify effective augmentation strategies [19].

Antipsychotic treatment is the first-line intervention in the management of schizophrenia and is recommended by major international guidelines [20]. However, approximately 20–30% of patients develop treatment-resistant schizophrenia (TRS) [21,22]. Treatment-resistant schizophrenia is generally defined as the persistence of clinically significant psychotic symptoms despite at least two adequate trials of different antipsychotic medications administered at therapeutic doses for an adequate duration, with confirmed treatment adherence [19,23]. Therefore, Clozapine is considered the gold-standard treatment for TRS and remains the only antipsychotic with established superiority in this patient population [23].

Nevertheless, approximately 40–60% of patients treated with clozapine continue to experience persistent psychotic symptoms, a condition commonly referred to as clozapine-resistant schizophrenia (CRS) [23,24,25]. Consequently, augmentation strategies aimed at improving therapeutic response have become increasingly important, with electroconvulsive therapy (ECT) representing one of the interventions supported by the strongest evidence for efficacy in clozapine-resistant schizophrenia [26,27].

Currently, electroconvulsive therapy is regarded as one of the most effective biological treatments in psychiatry [28,29,30]. Since its introduction into clinical practice more than eight decades ago, the procedure has undergone continuous refinement [28,30]. Modern ECT differs substantially from earlier methods, as it involves short-acting general anesthesia [31], neuromuscular blockade [30], individualized electrical stimulus dosing, continuous electroencephalographic (EEG) monitoring, and standardized safety protocols [28,29,30,32,33]. These advances have contributed to improved efficacy, enhanced tolerability, and a marked reduction in the incidence of serious adverse events [28,29,30,32].

International guidelines recommend the use of ECT in several clinical situations associated with schizophrenia, including treatment-resistant schizophrenia, catatonia, severe psychotic exacerbations requiring rapid symptom control, suicidality, refusal of food or fluids, and cases in which pharmacological treatment is insufficient or cannot be safely administered [30,34,35]. Numerous randomized clinical trials, systematic reviews, and meta-analyses have shown that ECT, particularly when combined with antipsychotic medication, leads to greater improvement in psychotic symptoms compared with pharmacotherapy alone [36,37,38,39,40]. Beneficial effects appear to be more pronounced for positive psychotic symptoms such as hallucinations, delusions, conceptual disorganization, agitation, and psychomotor excitement [36,37,38,39,40]. In contrast, findings regarding negative symptoms and cognitive functioning remain less consistent across studies [35,37,38,39,40].

Although the therapeutic efficacy and safety of modern ECT have been well established, its integration into routine psychiatric practice remains limited [28,30]. Access to contemporary ECT is frequently constrained by the need for specialized multidisciplinary teams, dedicated infrastructure, trained personnel, and institutional resources, while persistent stigma and misconceptions continue to hinder its wider acceptance [28,30,41,42]. Consequently, modern ECT remains underutilized despite being recommended by international guidelines [28,30]. In Romania, contemporary ECT is currently available in only a few specialized psychiatric centers, limiting access to this evidence-based intervention for many eligible patients [41,43]. Although real-world studies evaluating ECT have been published, reports from healthcare systems where access to modern ECT is limited remain relatively uncommon, and clinical experience with individualized ECT protocols in Romania has been only sparsely documented [41,43]. In this context, data generated by specialized centers are important for improving the understanding of contemporary ECT practice and may support the broader implementation of modern ECT in routine psychiatric care [41,43].

The exact mechanisms through which ECT exerts its therapeutic effects are not yet fully understood [44,45]. However, available evidence suggests that its efficacy cannot be explained solely by the induction of a generalized seizure [44,45,46]. Experimental studies and neuroimaging investigations indicate that ECT induces complex neurobiological changes involving neurotransmitter systems, neuroplasticity, functional brain connectivity, neuroendocrine regulation, inflammatory processes, and cerebral perfusion [45,46,47,48,49]. Proposed mechanisms include increased expression of brain-derived neurotrophic factor (BDNF), modulation of dopaminergic and glutamatergic neurotransmission, normalization of dysfunctional frontotemporal networks, and enhancement of synaptic plasticity [45,46,47,48,49]. These findings support the view that ECT is a complex neuromodulatory intervention capable of reorganizing dysfunctional neural circuits implicated in schizophrenia [46,47,48,49].

An essential component of contemporary ECT practice is the individualized determination of the seizure threshold, which enables stimulus dosing to be tailored to each patient’s physiological characteristics [28,50]. This approach optimizes therapeutic efficacy while minimizing unnecessary exposure to high-intensity electrical stimulation and reducing the risk of cognitive adverse effects [28,50,51,52]. Seizure threshold varies considerably between individuals and is influenced by multiple demographic, biological, and treatment-related factors, including age, sex, electrode placement, anesthetic agents, concomitant psychotropic medication, and prior ECT exposure [50,51,53]. Consequently, seizure threshold-based stimulus dosing has become a fundamental principle of evidence-based ECT practice [28,50,51,53].

Beyond the individualized determination of seizure threshold, the characteristics of the induced seizure have emerged as important factors influencing the therapeutic response to electroconvulsive therapy. While an adequate generalized seizure is necessary for the therapeutic effect of ECT, seizure duration requires careful monitoring. A seizure continuing for approximately two minutes or longer should prompt clinical management because of the potential for further progression, including the rare development of status epilepticus [28,43,54,55]. For this reason, seizure duration remains an essential parameter for monitoring and optimizing ECT [28,55]. However, increasing evidence suggests that seizure duration alone is an insufficient predictor of clinical response. Whereas seizure quality, reflected by electroencephalographic morphology, postictal suppression, and other neurophysiological markers, may provide a more comprehensive assessment of the relationship between seizure characteristics and therapeutic outcomes [28,43,55,56,57,58,59]. Furthermore, both therapeutic efficacy and the risk of cognitive adverse effects are influenced by the relationship between the administered electrical stimulus and the individual seizure threshold, supporting the use of personalized stimulus dosing in contemporary ECT practice [28,55,56,58,59]. Nevertheless, the clinical relevance of individual seizure quality parameters remains incompletely understood, and available evidence has been inconsistent.

Treatment outcomes following electroconvulsive therapy are influenced not only by patient-related factors but also by several technical aspects of treatment administration, including electrode placement, pulse width, stimulus frequency, stimulus duration, and electrical dose [28,32,58,59,60,61]. Bilateral electrode placement is generally associated with a more rapid and pronounced improvement in psychotic symptoms; however, this advantage is accompanied by a higher incidence of transient cognitive adverse effects. In contrast, unilateral ECT is typically associated with a more favorable cognitive profile, although higher stimulus intensities relative to the individual seizure threshold are often required to achieve comparable clinical efficacy [32,60,61]. In addition, concomitant psychotropic medications—including antipsychotics, benzodiazepines, mood stabilizers, and lithium—may influence seizure threshold, seizure quality, and overall treatment response [28,62]. Careful consideration of these technical and pharmacological factors is therefore essential for optimizing individualized ECT protocols and maximizing the balance between therapeutic efficacy and safety [28,32,50,60,61,62].

Clinical outcomes following electroconvulsive therapy are commonly evaluated using standardized psychopathological rating scales. The Positive and Negative Syndrome Scale (PANSS) is the most widely used instrument for assessing symptom severity in schizophrenia and remains the reference standard in both clinical practice and research [63,64]. Because ECT primarily targets acute psychotic manifestations, the PANSS positive subscale (PANSS-P) is particularly relevant for evaluating changes in delusions, hallucinations, conceptual disorganization, excitement, grandiosity, suspiciousness, and hostility [63,64,65]. However, improvements in psychotic symptoms should be interpreted alongside broader indicators of recovery. The Mini-Mental State Examination (MMSE) is widely used to assess global cognitive functioning and to monitor potential cognitive adverse effects associated with ECT, whereas the Global Assessment of Functioning (GAF) scale provides an overall measure of psychological, social, and occupational functioning [62,66,67].

Despite the growing body of evidence supporting the efficacy of ECT in schizophrenia, several clinically relevant questions remain unresolved. Most previous studies have focused primarily on global measures of psychopathology, such as the PANSS total score or the Brief Psychiatric Rating Scale (BPRS), whereas considerably less attention has been devoted to symptom-specific outcomes, cognitive performance, functional recovery, and the influence of procedural treatment characteristics on clinical response [36,37]. In addition, available evidence has been derived predominantly from randomized clinical trials or studies involving treatment-resistant schizophrenia, while relatively few naturalistic investigations have examined the interaction between individualized seizure threshold titration, stimulation parameters, seizure characteristics, and concomitant psychotropic medication in routine clinical practice [62]. Furthermore, the relationship between seizure threshold, seizure quality, and longitudinal changes in positive psychotic symptoms assessed using the PANSS positive subscale (PANSS-P) remains incompletely understood [54,62].

The aim of this retrospective naturalistic study was to describe the clinical outcomes of electroconvulsive therapy administered using individualized seizure threshold titration in patients with schizophrenia. In addition, treatment delivery was characterized by documenting stimulation parameters, seizure threshold, seizure characteristics, electrode placement, concomitant psychotropic medication, and patient characteristics. These variables were used to contextualize observed clinical outcomes without attempting to establish causal or predictive relationships. The primary outcome was the longitudinal change in PANSS-P score. Secondary outcomes included changes in MMSE and GAF scores, treatment response rates, and associations between clinical outcomes and seizure threshold, seizure characteristics, stimulation parameters, electrode placement, concomitant psychotropic medication, and selected patient characteristics.

## 2. Materials and Methods

### 2.1. Study Design and Setting

The present study followed a retrospective, naturalistic observational design and was conducted at the Department of Electroconvulsive Therapy of the Dr. Gh. Preda Clinical Psychiatric Hospital in Sibiu, within the Scientific Research Collective in Neurosciences. The study population consisted of consecutive patients diagnosed with treatment-resistant schizophrenia who underwent an acute course of electroconvulsive therapy (ECT). So, all the medical records of consecutive patients diagnosed with schizophrenia-spectrum disorders who underwent electroconvulsive therapy (ECT) between March 2022 and December 2025 (representing a three-and-a-half-year observation period) were reviewed. As a naturalistic investigation, all treatment-related decisions, including electrode placement, stimulus dosing, and concomitant psychotropic medication, reflected routine clinical practice and were based on individual clinical judgment rather than a standardized research protocol. All patients received ECT according to standard institutional protocols and current international recommendations. No modifications to treatment strategy were made for research purposes.

### 2.2. Participants

Participants were eligible for inclusion if they were 18 years of age or older and had a diagnosis of treatment-resistant schizophrenia according to the International Classification of Diseases, Tenth Revision (ICD-10; F20.x). Eligible patients had completed an acute course of electroconvulsive therapy (ECT), with the initial seizure threshold (IST) established during the first treatment session using a standardized dose-titration procedure. To ensure complete longitudinal evaluation, only patients with available pre- and post-treatment clinical assessments were included. Clinical outcome measures comprised the Positive and Negative Syndrome Scale (PANSS), the Mini-Mental State Examination (MMSE), and the Global Assessment of Functioning Scale (GAF). In addition, detailed records of motor and electroencephalographic (EEG) seizure duration for each ECT session, together with documentation of technical stimulation parameters and concomitant antipsychotic and adjunctive psychotropic medication throughout the treatment course, were required.

Patients receiving continuation or maintenance ECT were not included in the primary efficacy analyses because the objectives of continuation treatment differ from those of an acute therapeutic course. Additional exclusion criteria comprised incomplete clinical or ECT-related documentation, premature discontinuation of the acute ECT course before post-treatment assessment, or the presence of neurological or severe medical conditions with the potential to substantially influence psychiatric, cognitive, or functional outcomes. Patients who withdrew informed consent during treatment or who were subsequently admitted under involuntary hospitalization procedures were also excluded.

The study screening process identified 26 ECT courses performed in patients with treatment-resistant schizophrenia for whom baseline Positive and Negative Syndrome Scale–Positive Subscale (PANSS-P) assessments were available. Six continuation or maintenance ECT courses were excluded because the present study focused exclusively on the effectiveness of acute treatment. Of the remaining 20 acute ECT courses, three were not included in the primary outcome analyses because post-treatment PANSS-P assessments were unavailable for three acute ECT courses, as patients withdrew consent before completing the follow-up evaluation. Consequently, these cases were excluded from further analyses, precluding a descriptive comparison with the final study cohort.

The final study cohort comprised 17 acute ECT courses, 16 patients with schizophrenia, and one patient with schizoaffective disorder. Each of the 17 acute ECT courses included in the final analysis corresponded to a distinct patient; thus, no patient contributed more than one ECT course to the final analytical sample.

### 2.3. Ethical Considerations

The study was performed in accordance with the ethical principles outlined in the Declaration of Helsinki and received approval from the Ethics Committee of the Dr. Gh. Preda Clinical Psychiatric Hospital. Considering the retrospective nature of the study based on anonymized clinical records, patients’ consent for the use of their clinical data for research purposes had been obtained upon hospital admission, in line with institutional requirements.

### 2.4. Electroconvulsive Therapy Procedure

Electroconvulsive therapy was administered using a brief-pulse (bilateral (BL)) or ultrabrief (right unilateral (RUL)), constant-current stimulation system according to standard clinical practice. Treatments were performed under general anesthesia two to three times per week, with treatment frequency individualized according to clinical status, therapeutic response, and tolerability. General anesthesia was induced with intravenous propofol at a dose determined by the attending anesthesiologist, and neuromuscular relaxation was achieved with succinylcholine. Continuous electrocardiography, non-invasive blood pressure monitoring, and pulse oximetry were used throughout each session.

Seizure-threshold titration was performed progressively according to the device-specific titration protocol and the technical stimulation algorithm provided by the manufacturer. The initial stimulation level varied between patients, with stimulus level 2 representing the lowest starting level used in this cohort. At this level, the delivered charge was 19.2 mC for the 0.3-ms ultrabrief-pulse protocol and 48 mC for the 1.0-ms brief-pulse protocol [28]. If an adequate seizure was not elicited, stimulation was increased stepwise by advancing to the next level of the predefined titration table. An adequate seizure was operationally defined as an electrographic (EEG) seizure lasting at least 15 s [28]. The lowest stimulus dose that elicited an adequate EEG seizure was considered the individual seizure threshold and served as the reference for subsequent suprathreshold treatment dosing. In the absence of an observable motor response, seizure adequacy was assessed based on EEG activity; therefore, the absence of a motor seizure alone did not prompt restimulation when an adequate electrographic seizure was present [28]. If the EEG seizure duration was <15 s, stimulation was repeated at a higher dose according to the device-specific titration protocol [28,57].

This threshold served as the reference for subsequent suprathreshold stimulation, with treatment parameters adjusted according to electrode placement and clinical evolution. Right unilateral ECT was administered using the ultrabrief-pulse (0.3 ms) protocol, whereas bilateral ECT was administered using the brief-pulse (1.0 ms) protocol, with stimulation parameters subsequently adjusted according to the individualized seizure threshold and the corresponding device-specific dosing protocol, and it was selected individually by the treating psychiatrist based on symptom profile, previous treatment response, and clinical judgment. The total number of sessions and duration of the ECT course were similarly individualized and continued until satisfactory clinical improvement was achieved or further treatment was considered unlikely to provide additional benefit.

Seizure activity was assessed during each session using both motor and electroencephalographic (EEG) monitoring. Motor seizure duration was determined using the movement sensor integrated into the ECT device together with direct clinical observation, whereas EEG seizure duration was defined as the interval from onset to termination of ictal EEG activity recorded by the device. Both measures were documented in seconds for each session, and mean motor and EEG seizure durations across the entire ECT course were calculated for each patient. These measures were analyzed descriptively to characterize seizure quality and treatment delivery and to support interpretation of clinical outcomes, rather than to assess predictive or causal relationships.

### 2.5. Concomitant Pharmacological Treatment

All patients remained on pharmacological treatment during the ECT course as part of routine clinical care. Information on concomitant psychotropic medication was obtained from the medical records and classified into antipsychotics, mood stabilizers/anticonvulsants, and benzodiazepines, according to individual clinical needs. Concomitant psychotropic pharmacotherapy remained unchanged during the acute ECT courses included in the analysis to reduce potential confounding of treatment outcomes.

### 2.6. Clinical Assessment

Patients were evaluated at two predefined study visits: baseline, before the initiation of the electroconvulsive therapy (ECT) course, and the post-treatment assessment, conducted 48–72 h after completion of the acute ECT course to avoid evaluation during the immediate postictal period. All clinical assessments were performed by experienced clinicians routinely involved in patient care.

#### 2.6.1. Positive Psychotic Symptoms

The severity of positive psychotic symptoms was assessed using the Positive and Negative Syndrome Scale—Positive Subscale (PANSS-P), which comprises seven items covering delusions, conceptual disorganization, hallucinatory behavior, excitement, grandiosity, suspiciousness/persecution, and hostility. Treatment-related changes were examined by comparing PANSS-P total scores recorded at baseline and after completion of the acute ECT course.

#### 2.6.2. Global Functioning

Overall functioning was assessed using the Global Assessment of Functioning Scale (GAF), which measures psychological, social, and occupational functioning. GAF scores recorded at baseline and after completion of the acute ECT course were compared to assess changes in global functioning. The GAF was selected as a standardized measure of overall functional status commonly used in psychiatric settings.

#### 2.6.3. Cognitive Assessment

Cognitive functioning was assessed using the Mini-Mental State Examination (MMSE), a clinician-administered screening instrument evaluating multiple cognitive domains, including orientation, attention, memory, language, and visuospatial abilities. Changes in MMSE scores between baseline and post-treatment assessments were used to evaluate the cognitive effects of the acute ECT course. The MMSE was selected because it was the standardized cognitive screening instrument routinely used in clinical practice during the study period and, consequently, represented the only cognitive measure available for retrospective analysis.

### 2.7. Concomitant Psychotropic Medication

Data on concomitant psychotropic medications were retrospectively extracted from medical records and medication administration charts. All patients remained on pharmacological treatment during the ECT course as part of routine clinical care. Pharmacotherapy at baseline was categorized into four groups: antidepressants, antipsychotics, mood stabilizers/anticonvulsants, benzodiazepines, and the number of psychotropic drug classes prescribed for each patient was also recorded. Mood stabilizers/anticonvulsants represented pre-existing adjunctive pharmacotherapy prescribed according to individual clinical indications and were not initiated as part of the ECT protocol. Medication type and dosage were reviewed over the course of acute ECT and compared with baseline regimens to identify treatment adjustments. Concomitant psychotropic pharmacotherapy remained unchanged throughout the acute ECT courses included in the analysis, thereby minimizing potential confounding related to medication changes during treatment. These data were included to characterize pharmacological treatment during ECT and to provide context for the interpretation of clinical outcomes.

### 2.8. Outcome Measures

#### 2.8.1. Primary Outcome

The primary study outcome was the change in the severity of positive psychotic symptoms following ECT, quantified as the difference between baseline and post-treatment Positive and Negative Syndrome Scale—Positive Subscale (PANSS-P) scores (ΔPANSS-P).

#### 2.8.2. Secondary Outcomes

Secondary outcome measures included changes in overall functioning, assessed with the Global Assessment of Functioning Scale (GAF); changes in cognitive performance, measured by the Mini-Mental State Examination (MMSE); treatment response; remission status; and the relationship between ECT treatment parameters and clinical outcomes. Specifically, the effects of the initial seizure threshold, seizure duration, electrode placement, and concomitant psychotropic medication on treatment response were explored.

Clinical response was defined as a ≥20% reduction in the Positive and Negative Syndrome Scale positive subscale (PANSS-P) score from baseline to post-treatment. Given the variability in response definitions across schizophrenia studies, additional exploratory analyses were performed using more stringent response thresholds of ≥30% and ≥50% improvement, consistent with previously used approaches for evaluating different degrees of symptomatic response [68,69]. A post-treatment PANSS-P score of ≤14 was used to identify patients with a low level of residual positive symptoms, as a complementary measure of treatment outcome. This criterion was distinguished from the consensus definition of symptomatic remission in schizophrenia, which incorporates severity thresholds across eight core PANSS items [70]. Pre- and post-ECT PANSS-P scores were compared using a two-sided Wilcoxon signed-rank test. The magnitude of change was estimated using the Hodges–Lehmann estimator with a two-sided 95% confidence interval.

### 2.9. Variables of Interest

#### 2.9.1. Demographic and Clinical Variables

Demographic and clinical variables included age, sex, marital status, place of residence, occupational status, the diagnosis of schizophrenia, psychiatric and somatic comorbidities, duration of illness, and previous exposure to electroconvulsive therapy (ECT).

#### 2.9.2. ECT-Related and Pharmacological Variables

ECT-related variables included electrode placement (unilateral versus bilateral), initial seizure threshold, pulse width, stimulus duration, stimulation frequency, total number of ECT sessions, and motor and electroencephalographic seizure durations. Pharmacological variables included the number of concomitant antipsychotic medications and the use of mood stabilizers during the ECT course.

## 3. Results

### 3.1. Study Population

The unit of analysis was the individual ECT treatment course. Over the 3.5-year study period, 26 treatment courses involving patients with schizophrenia-spectrum disorders were identified and screened for eligibility. Baseline psychopathology was assessed in all cases using the Positive and Negative Syndrome Scale Positive Subscale (PANSS-P).

**Figure 1:**
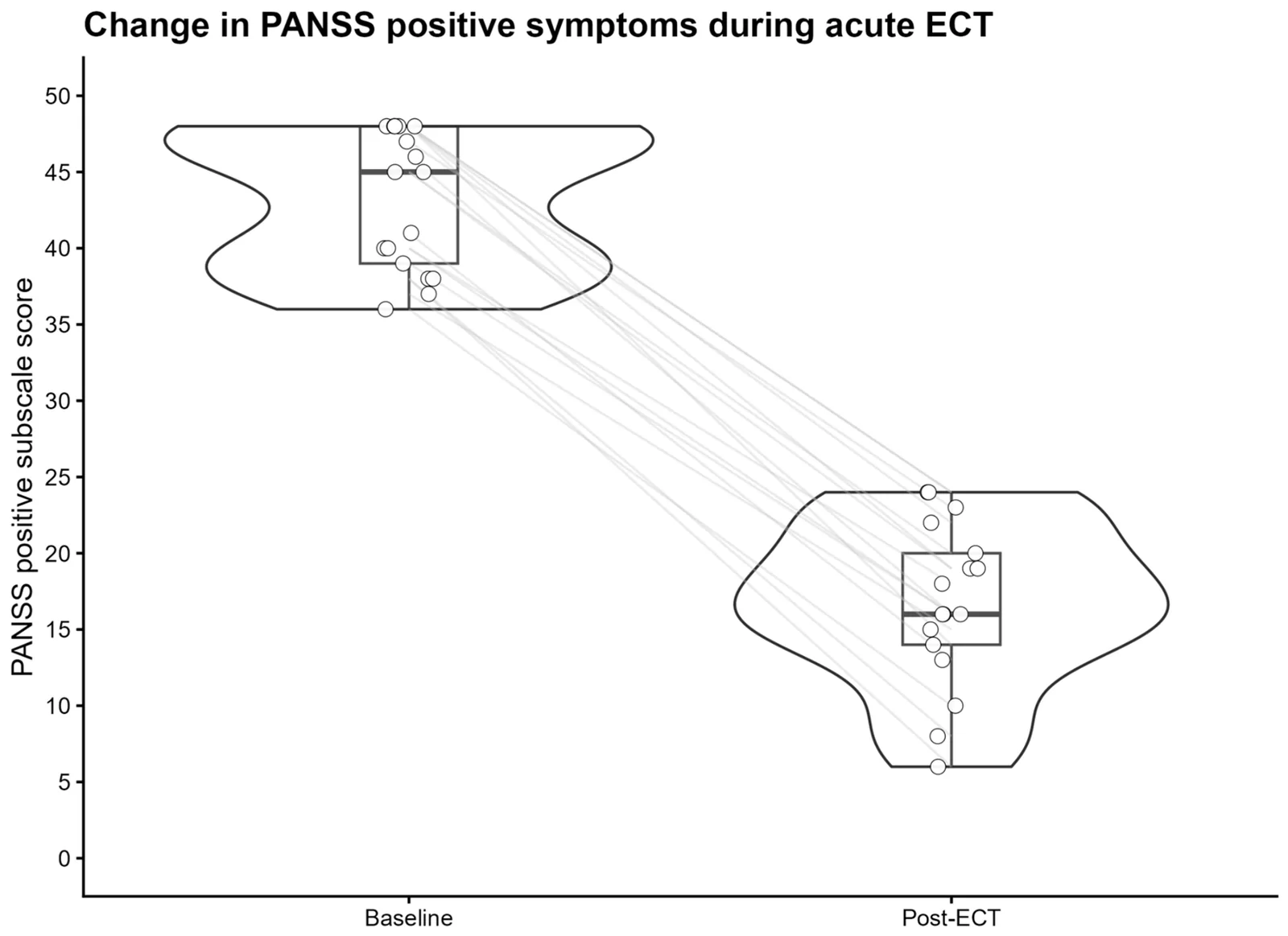
Change in positive symptom severity during acute ECT. Violin plots and boxplots show the distribution of Positive and Negative Syndrome Scale positive subscale (PANSS-P) scores at baseline and at the last available posttreatment assessment for acute ECT courses included in the primary analysis. Thin lines connect paired observations within courses, and individual points represent course-level scores.

Courses delivered as continuation or maintenance ECT (*n* = 6) were not included in the primary analyses because they represented a distinct therapeutic strategy from acute treatment. The remaining 20 acute treatment courses were subsequently assessed for the availability of paired baseline and post-treatment PANSS-P evaluations. Three courses were excluded owing to missing post-treatment assessments resulting from premature discontinuation of ECT or incomplete clinical follow-up. Accordingly, the primary analytic sample comprised 17 acute ECT treatment courses with evaluable paired baseline and post-treatment PANSS-P assessments (Figure 1). All data processing and statistical analyses were performed using R statistical software (version 4.5.1; R Core Team, 2025).

Within the acute analytic sample, the cohort was predominantly urban, unmarried, and socially inactive because of disability or retirement, with a substantial burden of somatic comorbidity. The median age was 37 years (IQR, 27–42), 9 of 17 courses (52.9%) involved male patients, and 14 of 17 (82.4%) were from urban settings. Most courses occurred in unmarried individuals (14/17 [82.4%]), and the majority were in patients classified as retired or receiving disability-related pension status (14/17 [82.4%]). Psychiatric comorbidity was documented in 4 courses (23.5%), and somatic comorbidity in 11 (64.7%). Baseline demographic and clinical characteristics are summarized in Table 1.

### 3.2. Clinical Outcomes

Positive psychotic symptom severity was lower post-ECT than at baseline (Figure 1). Median PANSS-positive score decreased from 45 (IQR, 39–48) at baseline to 16 (IQR, 14–20) after ECT. Consistent with our hypothesis, this within-course reduction was statistically significant on two-sided Wilcoxon signed-rank testing (V = 153, *p* < 0.001), with a Hodges–Lehmann estimate of 26 points (95% CI, 24–28), indicating a marked decrease in positive psychotic symptom severity during the acute ECT course.

**Figure 2:**
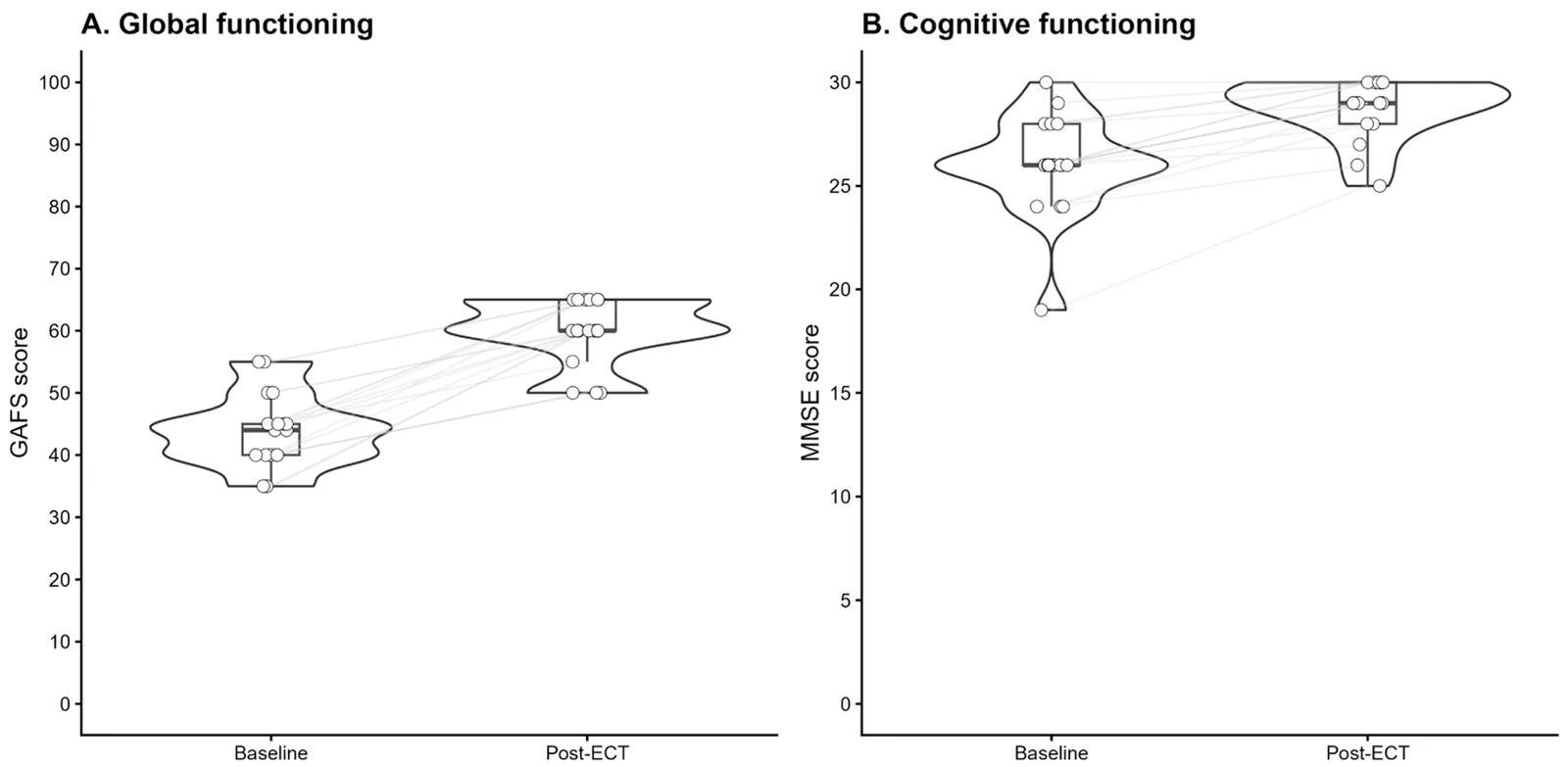
Changes in global functioning (**A**) and cognitive functioning (**B**). Violin-boxplots show the distribution of GAF and MMSE scores at baseline and post-ECT for the 17 acute ECT courses. Thin lines connect paired observations within individual courses. Post-treatment points are colored according to the degree of clinical improvement.

Changes in secondary clinical measures were consistent with improved global functioning and relative stability of cognitive performance, but were interpreted descriptively given the modest sample size and observational design. Median GAF score increased from 44 (IQR, 40–45) at baseline to 60 (IQR, 60–65) after ECT, whereas median MMSE score changed only modestly, from 26 (IQR, 26–28) to 29 (IQR, 28–30) (Figure 2).

**Figure 3:**
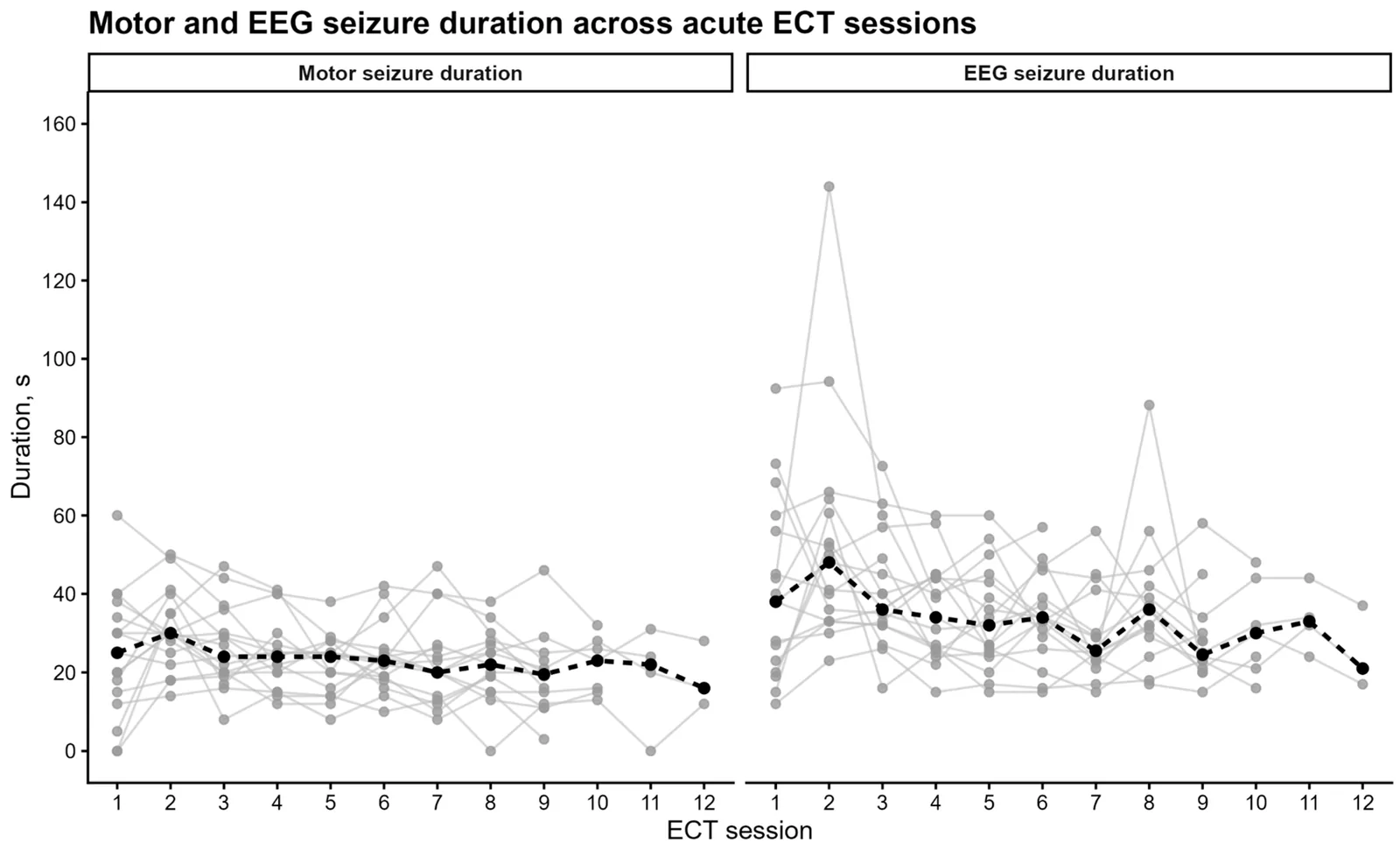
Motor and EEG seizure duration across acute ECT sessions. Thin gray lines connect individual acute ECT courses; points are colored according to the PANSS-P improvement category. The dashed black line and dots represent the overall median at each session. Because the number of courses without clinically meaningful improvement was small, visual comparisons between improvement categories are descriptive only.

The session-level expression of induced seizures is shown in Figure 3. Across acute ECT courses, both motor and EEG seizure durations demonstrated substantial within-course and between-course variability over successive treatment sessions. Motor seizure durations were generally shorter and clustered more tightly, whereas EEG seizure durations were longer overall and showed greater dispersion, particularly in the earlier sessions. Visual inspection of the session-level medians suggested some fluctuation across the treatment course, but no simple monotonic increase or decrease in seizure duration was apparent. Overall, these data indicate that seizure expression during acute ECT was heterogeneous across patients and sessions, with EEG seizure duration consistently exceeding motor seizure duration. Given the modest sample size, these findings were interpreted descriptively.

The technical characteristics of the acute ECT courses reflected individualized seizure threshold–guided treatment delivered under routine clinical practice (Table 2). Right unilateral and bilateral electrode placement were used with similar frequency, whereas stimulation parameters were adjusted according to individual seizure threshold titration. Concomitant antipsychotic treatment was maintained throughout all treatment courses, with most patients receiving combination antipsychotic therapy, while approximately two-thirds also received mood stabilizers or anticonvulsants. Antidepressant treatment was uncommon, and no modifications to psychotropic medication were introduced during the acute ECT course.

Taken together, these findings indicate that in this naturalistic cohort of patients with treatment-resistant schizophrenia, seizure threshold-titrated ECT was associated with substantial improvement in positive psychotic symptoms and overall functioning. Global cognitive performance, as measured by the MMSE, showed no deterioration following the acute ECT course. Owing to the limited sample size and observational design, these observations remain exploratory; however, they suggest a potential role for individualized ECT in the acute management of treatment-resistant schizophrenia.

## 4. Discussion

This 3.5-year retrospective study evaluated the clinical outcomes of seizure threshold-titrated electroconvulsive therapy in patients with schizophrenia-spectrum disorders, with particular emphasis on positive psychotic symptom severity, global functioning, cognitive performance, and treatment-related seizure characteristics. Several clinically relevant findings emerged. First, acute ECT was associated with a marked reduction in positive psychotic symptom severity, with median PANSS-P scores decreasing from 45 (IQR, 39–48) at baseline to 16 (IQR, 14–20) after ECT. The Hodges–Lehmann estimate indicated a paired reduction of 26 points, and the within-course change was statistically significant (*p* < 0.001), supporting a substantial decrease in positive symptom burden during acute treatment. Second, symptomatic improvement was accompanied by a marked increase in global functioning, with median GAF scores increasing from 44 to 60. Third, global cognitive screening performance showed no deterioration, with median MMSE scores increasing from 26 to 29. Finally, motor and EEG seizure durations demonstrated substantial within- and between-course variability, without a clear monotonic trajectory across successive ECT sessions.

The pronounced reduction in positive psychotic symptoms observed in the present cohort is consistent with the growing body of evidence supporting ECT as an augmentation strategy in schizophrenia, particularly in patients with severe, persistent, or treatment-resistant symptoms [36,71,72]. Given the recognized clinical heterogeneity of treatment-resistant schizophrenia and the challenges in defining and managing inadequate antipsychotic response [73], our findings further suggest a potential role for adjunctive ECT when clinically significant positive symptoms persist despite ongoing pharmacological treatment, including in selected patients with clozapine-resistant illness [26,36,71,72].

The magnitude of positive symptom improvement observed in our cohort is broadly consistent with findings from controlled studies of adjunctive ECT. In a meta-analysis of 11 randomized controlled trials including 818 patients with treatment-resistant schizophrenia, Zheng et al. demonstrated that ECT combined with non-clozapine antipsychotic medication was superior to antipsychotic monotherapy for overall symptomatic improvement, with significant benefits also observed for PANSS positive and general psychopathology subscale scores [36]. Adjunctive ECT was additionally associated with higher study-defined response and remission rates, with differences between ECT augmentation and antipsychotic monotherapy becoming apparent during the early phase of treatment [36]. Recent meta-analytic evidence further suggests that adjunctive ECT may promote a more rapid symptomatic response in schizophrenia compared with antipsychotic treatment alone [74]. This potential for earlier improvement may be particularly relevant in severely symptomatic hospitalized patients, for whom rapid control of positive psychotic symptoms is clinically important.

An additional methodological strength of the present study is the use of the Positive and Negative Syndrome Scale Positive Subscale (PANSS-P) as the primary symptomatic outcome measure. Whereas many previous ECT studies in schizophrenia have relied predominantly on total PANSS scores, the PANSS-P specifically captures core positive symptoms, including delusions, hallucinatory behavior, conceptual disorganization, excitement, grandiosity, suspiciousness/persecution, and hostility [63,64]. This symptom-domain approach is particularly relevant in the context of acute ECT, where reduction of severe positive psychotic symptoms may represent an important therapeutic objective, whereas negative symptoms and broader psychopathology may reflect more heterogeneous and persistent dimensions of illness and follow different trajectories during a relatively short treatment course [34,37,64].

This distinction is clinically relevant because changes in total PANSS scores do not necessarily reflect comparable improvement across individual symptom domains. For example, improvement in overall psychopathology may occur without a proportional reduction in core positive symptoms, while marked improvement in hallucinations, delusions, or thought disorganization may coexist with persistent symptoms in other domains. Accordingly, the use of PANSS-P allowed a more focused characterization of positive symptom change in the present cohort and may provide a clinically informative outcome for studies specifically examining symptom-domain responses to ECT.

Evidence supporting ECT augmentation also extends to more refractory forms of schizophrenia. ECT augmentation of clozapine has been investigated in patients with clozapine-resistant schizophrenia, with meta-analytic evidence supporting additional symptomatic benefit in selected patients [72]. Moreover, an international expert consensus from the Treatment Response and Resistance in Psychosis Working Group identified ECT augmentation as a potential strategy for persistent positive symptoms despite adequate clozapine treatment [26]. Direct comparison between these studies and our findings should nevertheless be made cautiously because of differences in patient selection, degree of treatment resistance, concomitant pharmacotherapy, ECT technique, outcome definitions, and study design. Accordingly, the present findings should be considered complementary real-world evidence rather than a direct estimate of comparative ECT efficacy.

An important characteristic of the present study was the stability of concomitant pharmacological treatment during the acute ECT course. All patients were receiving antipsychotic medication at baseline, most received combination antipsychotic therapy, and no modifications to the psychotropic regimen were introduced during the included acute courses. This pattern is consistent with the predominant contemporary use of ECT in schizophrenia as an augmentation strategy alongside ongoing antipsychotic pharmacotherapy [26,36,71]. Although the observational design does not permit the observed improvement to be attributed exclusively to ECT, the absence of major medication changes during treatment reduces one potential source of within-course confounding and strengthens the temporal association between ECT administration and the reduction in positive symptom severity.

The improvement in positive psychotic symptoms was accompanied by a substantial increase in global functioning, with median GAF scores increasing from 44 at baseline to 60 following treatment. This finding provides additional clinical context to the reduction in PANSS-P scores, as symptomatic improvement and functional recovery represent related but not identical dimensions of outcome in schizophrenia. Previous studies have demonstrated significant associations between PANSS symptom severity and GAF scores, including longitudinal relationships between changes in psychopathology and changes in global functioning [74,75]. Nevertheless, these relationships are only partial, reflecting the fact that functional outcome in schizophrenia is influenced by multiple dimensions, including positive and negative symptoms, cognition, social functioning, and illness-related disability [74,75,76].

The concurrent improvement in PANSS-P and GAF scores in the present cohort therefore suggests that the clinical changes observed during acute ECT were accompanied by broader improvement in overall functioning rather than being restricted to positive symptom ratings alone. However, this finding should be interpreted cautiously. The GAF combines symptom severity with psychological, social, and occupational functioning within a single global score and therefore does not provide an independent domain-specific measure of functional recovery [74,76]. Consequently, the increase in GAF observed in our cohort should be interpreted as evidence of improvement in overall clinical functioning rather than as proof of recovery in specific occupational, interpersonal, or independent-living domains. Furthermore, given the retrospective design and absence of a control group, a causal relationship between ECT and functional improvement cannot be established.

Global cognitive screening performance showed no deterioration during the acute treatment course, with median MMSE scores increasing from 26 to 29. This observation is clinically relevant because cognitive adverse effects remain an important consideration in ECT treatment. However, the modest increase in MMSE score should not be interpreted as evidence of cognitive enhancement following ECT, nor does the absence of MMSE deterioration exclude more subtle treatment-related cognitive effects. The MMSE is a global screening instrument and has limited sensitivity for detecting domain-specific changes, particularly in autobiographical and anterograde memory. The present findings should therefore be interpreted as indicating no detectable deterioration in global cognitive screening performance rather than demonstrating the absence of ECT-related cognitive effects.

This cautious interpretation is consistent with schizophrenia-specific evidence regarding cognitive outcomes following ECT [77,78]. A systematic review examining objective cognitive outcomes after ECT in treatment-resistant schizophrenia found no consistent evidence of persistent deterioration across several cognitive domains, including global cognition, attention, language, visuospatial functioning, and executive functioning, whereas findings regarding memory were more heterogeneous [77]. A separate systematic review examining stimulus parameters and electrode placement similarly concluded that schizophrenia-specific evidence remains limited, although right unilateral and bifrontal approaches may offer cognitive advantages over bitemporal ECT in some circumstances [78]. Thus, the stability of MMSE scores observed in the present cohort is reassuring but should be considered in light of the instrument’s limited sensitivity and the absence of comprehensive neuropsychological assessment.

Electrode placement may be particularly relevant to the balance between therapeutic efficacy and cognitive tolerability. In the present cohort, right unilateral and bilateral electrode placements were used with similar frequency, reflecting individualized clinical decision-making. In a randomized naturalistic comparison of right unilateral, bifrontal, and bitemporal ECT in schizophrenia, symptomatic improvement was observed across treatment groups, although improvement in positive symptoms was less pronounced with right unilateral treatment than with bifrontal or bitemporal ECT, while bitemporal treatment was associated with greater impairment on several memory measures [79]. Naturalistic evidence has likewise demonstrated clinically relevant symptomatic improvement with ECT while emphasizing the potential importance of electrode configuration and stimulus parameters for both therapeutic and cognitive outcomes [80]. Because electrode placement was clinically individualized rather than randomized in the present study, and because the sample was small, our data do not permit reliable comparisons of efficacy or cognitive outcomes between electrode configurations.

The individualized seizure threshold-guided approach used in the present cohort is also relevant when interpreting the technical ECT parameters. Seizure threshold varies substantially between individuals and may be influenced by age, electrode placement, treatment number, concomitant medications, and other patient- and treatment-related factors [81,82]. Stimulus titration therefore provides a means of estimating the individual seizure threshold and tailoring subsequent stimulus dosing rather than applying uniform electrical parameters across patients. Previous work has demonstrated considerable interindividual variability in seizure threshold during ECT, supporting individualized dosing approaches [81,82].

Motor and EEG seizure durations demonstrated considerable variability both between patients and across successive treatment sessions. EEG seizure duration was generally longer than motor seizure duration, while neither measure demonstrated a simple monotonic increase or decrease over the acute treatment course. These observations are consistent with the broader understanding that seizure duration alone is an incomplete marker of ECT treatment adequacy. The therapeutic properties of an induced seizure depend on multiple technical and physiological factors, including stimulus dose relative to seizure threshold, electrode placement, and ictal EEG characteristics [58,59,83,84]. Accordingly, variability in seizure duration should not necessarily be interpreted as evidence of inconsistent treatment quality, particularly when ECT is delivered using individualized seizure threshold-guided dosing.

The relationship between seizure duration and clinical response in schizophrenia remains incompletely defined. Some observational evidence has suggested that EEG seizure duration may differ between ECT responders and nonresponders, whereas other studies have failed to demonstrate a clear relationship between average seizure duration and symptomatic improvement [85,86]. These heterogeneous findings suggest that seizure duration may provide clinically relevant information in some circumstances but is unlikely to function as a stand-alone surrogate for therapeutic efficacy. The substantial within- and between-course variability observed in the present study therefore reinforces the need to interpret seizure duration alongside clinical response and other measures of seizure quality rather than in isolation.

The number of ECT sessions administered in the present cohort was also broadly compatible with previous real-world experience in schizophrenia. Most acute courses consisted of 9–12 sessions, with nine sessions representing the most frequently administered course length. Previous observational evidence has demonstrated a progressive accumulation of response across an acute ECT course, with additional patients achieving clinically meaningful improvement after six, nine, and twelve treatment sessions [85]. These observations support an individualized approach to determining acute-course duration rather than reliance on a universally fixed number of treatments. Recent meta-analytic evidence examining the temporal course of response similarly supports the possibility that adjunctive ECT may accelerate symptomatic improvement when combined with antipsychotic pharmacotherapy [74].

Several limitations should be considered when interpreting the present findings. First, the retrospective and observational design precludes causal inference, and the absence of a control group prevents separation of the effects of ECT from other time-dependent clinical factors. Second, the primary analytic sample comprised only 17 acute ECT courses, resulting in limited statistical power and precluding robust subgroup analyses according to electrode placement, medication regimen, demographic characteristics, or other potential predictors of response.

Third, analyses were based on 17 treatment courses from 17 distinct patients, with each course representing a separate treatment episode with its own baseline assessment. While observations were therefore independent at the patient level, the small sample size limited statistical precision and the ability to account for clinical heterogeneity.

Fourth, three patients who initiated treatment withdrew informed consent during the treatment course and were consequently excluded from the final analysis, as post-treatment PANSS-P assessments were unavailable. Their exclusion may have introduced attrition bias and should be considered when interpreting the treatment outcomes.

Fifth, cognitive outcome assessment relied on the MMSE, which is insufficiently sensitive to characterize subtle or domain-specific cognitive effects of ECT.

Sixth, the limited availability of ECT in Romania represents an important contextual limitation. At the time of the study, ECT was available in only two specialized centers nationwide, substantially restricting access to treatment and the number of patients available for inclusion [43]. This contributed to the small sample size and may have introduced referral or selection bias, as patients treated in specialized ECT centers may not be representative of the broader population eligible for ECT, thereby limiting the generalizability of our findings.

An additional limitation is that individual antipsychotic agents were not systematically recorded in the retrospective dataset, preventing assessment of their potential influence on seizure threshold and seizure characteristics.

Finally, the study focused primarily on positive symptoms; therefore, the findings cannot be generalized to negative symptoms, cognitive symptoms, or the broader multidimensional construct of symptomatic remission in schizophrenia.

Despite these limitations, the present study provides clinically relevant real-world evidence supporting individualized, seizure threshold-titrated ECT as an adjunctive treatment strategy for persistent positive symptoms in schizophrenia-spectrum disorders [36,71,73]. The variability in seizure expression highlights the need for systematic evaluation of stimulation parameters and seizure-quality measures, as seizure duration alone may be insufficient to characterize treatment adequacy or clinical response [81,82,83,84,85,86]. Future prospective studies should include larger cohorts and integrate standardized clinical outcomes with comprehensive assessment of ECT stimulation characteristics, seizure quality, cognitive outcomes, and longer-term follow-up to better determine how treatment-related and patient-specific factors influence therapeutic response.

## 5. Conclusions

In this naturalistic retrospective cohort, individualized seizure threshold-titrated ECT administered alongside stable antipsychotic pharmacotherapy was associated with a substantial reduction in positive psychotic symptoms and improvement in global functioning in patients with schizophrenia-spectrum disorders. Global cognitive screening performance showed no deterioration during the acute treatment course. Motor and EEG seizure characteristics showed considerable variability across patients and treatment sessions; however, these observations were descriptive and do not allow conclusions regarding their relationship with clinical response. Given the retrospective observational design and absence of a control group, no causal relationship between ECT and the observed clinical improvement can be established. Overall, these findings provide preliminary real-world evidence supporting further investigation of individualized ECT as an adjunctive strategy for persistent positive symptoms. Larger prospective controlled studies integrating standardized clinical and cognitive outcomes with systematic assessment of stimulation parameters and seizure-quality measures are needed to confirm these findings and clarify their relationship with therapeutic response.

## Figures and Tables

**Table 1:** Baseline demographic and clinical characteristics.

Characteristic	N = 17
Age, y, Median (Q1, Q3)	37 (27, 42)
Sex, *n* (%)	
Female	8 (47.1%)
Male	9 (52.9%)
Residence, *n* (%)	
Urban	14 (82.4%)
Rural	3 (17.6%)
Marital status, *n* (%)	
Married	1 (5.9%)
Unmarried	14 (82.4%)
Divorced	2 (11.8%)
Occupational/social category, *n* (%)	
Employed	3 (17.6%)
Retired	14 (82.4%)
Primary diagnosis, *n* (%)	
Schizoaffective disorder	1 (5.9%)
Schizophrenia	16 (94.1%)
Any psychiatric comorbidity, *n* (%)	4 (23.5%)
Any somatic comorbidity, *n* (%)	11 (64.7%)
ECT sessions, No., *n* (%)	
6	1 (5.9%)
7	1 (5.9%)
8	1 (5.9%)
9	8 (47.1%)
10	2 (11.8%)
11	1 (5.9%)
12	3 (17.6%)
Repeated admission during study period refers to repeated psychiatric hospitalization during the study period and does not indicate that a patient contributed more than one ECT course to the final analysis, *n* (%)	6 (35.3%)

Values are median (IQR) for continuous variables and n (%) for categorical variables. Acute ECT courses with evaluable pre/post PANSS-positive assessments were included. Continuation/maintenance courses and courses without evaluable posttreatment PANSS-positive data were excluded.

**Table 2:** ECT course characteristics and concomitant psychotropic treatment.

Characteristic	N = 17
Electrode placement, *n* (%)	
Right unilateral	8 (47.1%)
Bilateral	8 (47.1%)
Mixed	1 (5.9%)
Stimulus number at treatment initiation, *n* (%)	
2	11 (64.7%)
3	5 (29.4%)
5	1 (5.9%)
Pulse width at treatment initiation, ms, *n* (%)	
0.3	9 (52.9%)
1	8 (47.1%)
Frequency at treatment initiation, Hz, *n* (%)	
20	15 (88.2%)
25	1 (5.9%)
30	1 (5.9%)
Stimulus duration at treatment initiation, s, *n* (%)	
1.5	4 (23.5%)
2	7 (41.2%)
3	3 (17.6%)
4	1 (5.9%)
6	1 (5.9%)
8	1 (5.9%)
Any antidepressant at baseline, *n* (%)	3 (17.6%)
Antidepressants at baseline, No., *n* (%)	
0	14 (82.4%)
1	2 (11.8%)
2	1 (5.9%)
Any antipsychotic at baseline, *n* (%)	17 (100.0%)
Antipsychotics at baseline, No., *n* (%)	
1	4 (23.5%)
2	9 (52.9%)
3	1 (5.9%)
4	2 (11.8%)
5	1 (5.9%)
Any mood stabilizer/anticonvulsant at baseline, *n* (%)	11 (64.7%)
Psychotropic classes at baseline, No., *n* (%)	
1	5 (29.4%)
2	10 (58.8%)
3	2 (11.8%)

Values are median (IQR) for continuous variables and *n* (%) for categorical variables. Stimulation parameters reflect the setting at treatment initiation. Mixed courses indicate switching from right unilateral to bilateral placement. Continuation/maintenance courses and courses without evaluable posttreatment PANSS-positive assessments were excluded. Concomitant treatment was recorded as unchanged during ECT in all included courses.

## Data Availability

Anonymized data are available from the corresponding author upon reasonable request.
